# Incense smoke: clinical, structural and molecular effects on airway disease

**DOI:** 10.1186/1476-7961-6-3

**Published:** 2008-04-25

**Authors:** Ta-Chang Lin, Guha Krishnaswamy, David S Chi

**Affiliations:** 1 Department of Environmental Engineering, National Cheng Kung University, Tainan, Taiwan; 2 Sustainable Environment Research Center, National Cheng Kung University, Tainan, Taiwan; 3 Department of Internal Medicine, James H. Quillen College of Medicine, East Tennessee State University, Johnson City, TN, USA

## Abstract

In Asian countries where the Buddhism and Taoism are mainstream religions, incense burning is a daily practice. A typical composition of stick incense consists of 21% (by weight) of herbal and wood powder, 35% of fragrance material, 11% of adhesive powder, and 33% of bamboo stick. Incense smoke (fumes) contains particulate matter (PM), gas products and many organic compounds. On average, incense burning produces particulates greater than 45 mg/g burned as compared to 10 mg/g burned for cigarettes. The gas products from burning incense include CO, CO_2_, NO_2_, SO_2_, and others. Incense burning also produces volatile organic compounds, such as benzene, toluene, and xylenes, as well as aldehydes and polycyclic aromatic hydrocarbons (PAHs). The air pollution in and around various temples has been documented to have harmful effects on health. When incense smoke pollutants are inhaled, they cause respiratory system dysfunction. Incense smoke is a risk factor for elevated cord blood IgE levels and has been indicated to cause allergic contact dermatitis. Incense smoke also has been associated with neoplasm and extracts of particulate matter from incense smoke are found to be mutagenic in the Ames Salmonella test with TA98 and activation. In order to prevent airway disease and other health problem, it is advisable that people should reduce the exposure time when they worship at the temple with heavy incense smokes, and ventilate their house when they burn incense at home.

## Introduction

Encyclopedia Britannica states that incense was employed to counteract disagreeable odors, drive away demons, manifest the presence of gods, and to gratify gods. Incense burning has been practiced for centuries. Early Christian churches used incense in the Eucharistic ceremony, in which it symbolized the ascent of the prayers of the faithful and the merits of the saints. Later, incense was employed sporadically in the Church of England. Elsewhere in both Eastern and Western Catholic Christendom, its use during divine worship and during processions has been continuous [1]. In Asian countries where the Buddhism and Taoism are mainstream religions, such as China, Thailand, and Taiwan, incense burning is a daily practice.

**Figure 1:**
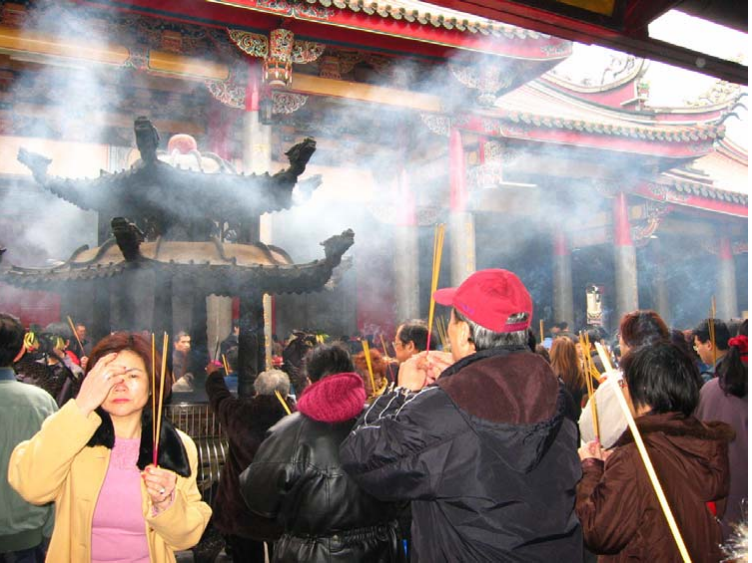
**Incense burning during Lunar New Year in the Long-Shang Temple in Taipei, Taiwan.** Apaprently, the dense incense smoke inflicted irritation in the eyes of a worshiper (photo by T. C. Lin).

In Taiwan, about half of its population (23 million) is Buddhist or Taoist. Most of them burn incense daily when they worship at home. The people in Taiwan also worship with incenses at temples regularly. In 2003, the Environmental Protection Agency in Taiwan reported that a total of 28.7 metric tons of incense was burned in 92 temples in Kao-Hsiong City [2]. It is equivalent to 0.86 kg/temple/day. Currently, there are 11,503 registered temples in Taiwan [3]. It is estimated that at least a total of 3,580 tons of incense is consumed yearly in the temples in Taiwan. During the Lunar New Year and other religious festivals, a huge amount of incense is burned in temples (Figure 1). If household incense burning is included, the incense consumption in Taiwan may even double or triple that estimated amount and it may indicate an environmental hazardous situation.

The air pollution in and around various temples has been documented [4-12]. The effects of incense smoke on airway disease and health also have been reported. This article will review: the nature of incenses and incense burning, pollutants emitted from incense burning, and effects of incense smoke on airway disease and health.

## The nature of incenses and incense burning

**Figure 2:**
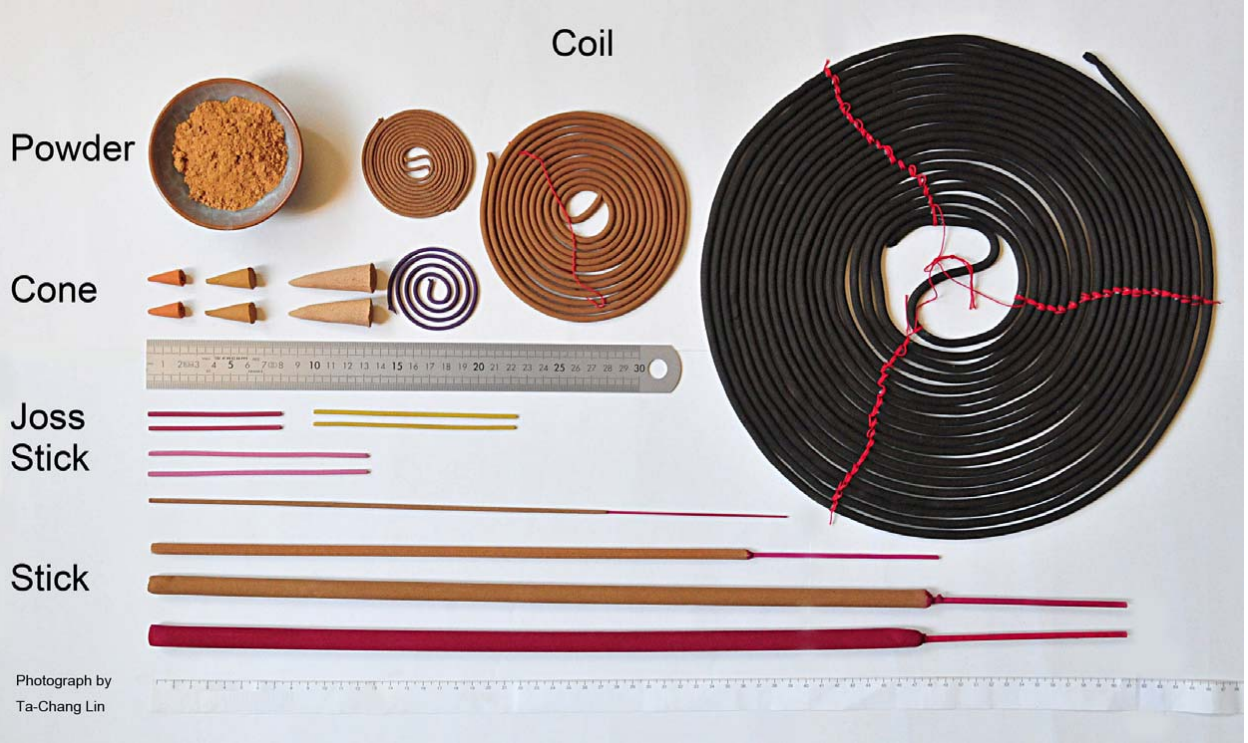
**Forms of incense.** Major forms of incense are shown, including powder, coil, cone, joss stick, and stick. (photo by T. C. Lin).

There are various forms of incenses, including sticks, joss sticks, cones, coils, powders, rope, rocks/charcoal, and smudge bundles [13]. The main difference between the first two forms is that the former has a slender bamboo base, onto which the mixture of incense ingredients is attached, while the latter is without a central base. Figure 2 shows five major forms of Asian incense, among them stick incense is the most popular in Taiwan.

Depending on its makers and local custom, incense sticks have several commercially available types, such as Chen Shan (Shan means incense), Gui Shan, Hsing Shan, Lao Shan, and Liao Shan. However, the physical characteristics of these incenses, such as length and diameter of the bamboo stick (average 39.5 and 0.4 cm, respectively), length and diameter of the incense coated part (average 28.5 and 2.7 cm, respectively), and weight of the whole stick (average 1.3 gm), are very similar [14]. While the exact content of incense sticks is a commercial secret, most incense is made from a combination of fragrant gums, resins, wood powders, herbs and spices.

A typical composition of stick incense consists of 21% (by weight) of herbal and wood powder, 35% of fragrance material, 11% of adhesive powder, and 33% of bamboo stick [15]. Herbal and wood powders used in incense making include Glycyrrhiza uralensis Fisch. (Leguminosae), Cinnamomum cassia Bl. (Lauraceae), Nardostachys chinensis Bastal. (Valerianaceae), Foeniculum vulgare Mill. (Umbelliferae), Rheum officinale Baill. (Polygonaceae), Radix Aucklandia. (Compositae), Asarum siebolidii Miq. (Aristolochiaceae), Magnolia liliiflora Desr. (Magnoliaceae), Eugenia caryophyllata Thumb. (Myrtaceae), and Ocimum basilicum L. (Labiatae) [15]. Some of these materials are also used in Chinese traditional medicine. Fragrance materials used in incense source from Lysimachia foenum-graecum. (Primulaceae), Juniperus chinensis L. var. Kaizuka Hort. (Cupressaceae), Liquidambar formosana Hance. (Hamamelidaceae), Santalum album L. (Santalaceae), Musk ambrette, musk ketone, and musk xylene. Adhesive Powder is from the bark of Machilus nanmu Hemsl. (Lauraceae). To make incenses, one end of a bamboo stick is first soaked in adhesive materials before it is coated with a mixture of fragrance, herbal and wood powders. This coating process is repeated two more times. Incenses are then dried under the sun.

Traditionally, incense burning usually involves three or more sticks simultaneously. It will take from 50 to 90 minutes to burn a stick of incense. When incense is burning, it emits smoke (fumes) containing particulate matter (PM), gas products and other organic compounds. Once the incense coating section has burned completely, the burning extinguishes itself at the tip of the bare bamboo part of the stick. The gas products from burning incense include CO, CO_2_, NO_2_, SO_2_, and others. Incense burning also produces volatile organic compounds, such as benzene, toluene, and xylenes, as well as aldehydes and polycyclic aromatic hydrocarbons (PAHs), which mostly are absorbed on particle matter.

## Major types of air pollutants in incense smoke and their toxicological effects

People who are exposed to incense fumes always inhale the whole complex mixture that contains particulate matter, gas products and many organic compounds. It is therefore difficult, if not impossible, to single out the health effects contributed by a certain component in the fumes. For example, there hasn't been any report about the ill effects on human health directly caused by the particles per se in the incense smoke.

Nevertheless, it's still helpful to know the composition of incense smoke in terms of types of pollutants and the corresponding toxicological effects – even though these cited effects were obtained from non-incense studies on air pollutants in general.

### 1. Particulate matter (PM)

From practical considerations of the health effects, air particulates are usually categorized according to how deep they can penetrate into the human respiratory system. Coarse particles are those greater than 10 μm in diameter. They are too large to enter the human respiratory system, hence causing no immediate threat. Particles less than 10 μm in diameter (PM_10_) pose a health concern because when inhaled they can accumulate in the respiratory system. Particles in the range 10 to 2.5 μm are known as the thoracic coarse particles (PM_10-2.5_) [16]. Particles less than 2.5 μm in diameter (PM_2.5_) are referred to as fine particles and are believed to pose the largest health risks because they can go as deep as the alveoli [17,18]. Particles less than 0.1 μm are called ultrafine particles [19].

Since people who are exposed to incense smoke always inhale a complex mixture of both gaseous and particulate products from the incense, it is difficult to single out the health effects of incense particles alone. So far, there hasn't been any report about the ill effects on human health directly caused by the particles per se in the incense smoke. Epidemiological studies have reported associations between air particulate matter (especially the fine particles) and several acute health effects, including mortality, hospital admissions, respiratory symptoms, and lung dysfunction [20-25]. The USEPA 2004 *Air Quality Criteria for Particulate Matter *conclusion states that PM_10-2.5 _exposure was associated with respiratory morbidity [26,27].

The combustion of incense, wood, cigarette, and candles is important or even major sources of residential indoor particulate matter, especially in the 2.5 μm size range and below [4-6,4,13,28-30]. Mannix et al. reported that burning incense could generate large quantities of PM. On average, it produces PM greater than 45 mg/g burned, as compared to 10 mg/g burned for the cigarettes [31]. Lin et al. measured 1,316 and 73 μg/m^3^, respectively, for the mean indoor and outdoor total suspended particulate (TSP) concentrations at one Taiwanese temple [7]. In a study of the indoor air pollution in Taiwan, Liao et al. [32] found that incense burning had size integrated source emission rates of 0.038 ± 0.026 particles/second. For indoor particles ranging from 0.5 to 5 μm, 62–92% is from indoor sources, including cooking, incense burning, and other residential activities. It is important to know that addition of calcium carbonate in incense can effectively suppress the particulate emission by as much as 40%; hence calcium carbonate may make the incense safer to use [14].

### 2. Gaseous emissions

#### 2.1. Carbon monoxide (CO)

Carbon monoxide is a colorless, odorless, tasteless, yet poisonous gas generally formed during incomplete combustion of organic substances, such as hydrocarbons, wood, incense, cigarette, and fossil fuels. CO combines with haemoglobin much more readily than oxygen, by a factor of 200–300, hence reduces the blood's capacity to transport oxygen. Inhalation of CO in low concentrations can cause headaches, dizziness, weakness and nausea, while high concentrations can be fatal [33].

#### 2.2. Sulfur dioxide (SO_2_) and nitrogen dioxide (NO_2_)

Health effects of exposures to sulfur dioxide, and nitrogen dioxide can include reduced work capacity, aggravation of existing cardiovascular diseases, effects on pulmonary function, respiratory illnesses, lung irritation, and alterations in the lung's defense system [34].

#### 2.3. Volatile organic compounds

Volatile organic compounds (VOCs) are chemicals that have low boiling points and therefore evaporate easily at room temperature. Common VOCs include benzene, toluene, xylenes, and isoprene. Acute symptoms of VOC exposures are: eye irritation/watering, nose irritation, throat irritation, headaches, nausea/vomiting, dizziness, and asthma exacerbation. Chronic symptoms of VOC exposure are: cancer, liver damage, kidney damage, central nervous system damage [35].

Löfroth et al. [28] found that smoking and incense burning generates CO, isoprene and benzene. Lee et al. [36] burned incense in a large environmental chamber. They found that, while the benzene and toluene levels recommended by the Indoor Air Quality Objectives for Office Buildings in Hong Kong (HKIAQO, 1999) are 16.1 and 1,092 μg/m^3^, respectively, the measured benzene concentrations of all tested incense were significantly higher than the standard.

#### 2.4. Aldehydes

Most materials produce aldehydes and ketones during combustion. Burning incense is also known to generate aerosols and formaldehyde [37,36,40]. Lin and Tang investigated the content of particulates in Chinese incense smoke and found that acrolein, formaldehyde and acetaldehyde were predominantly adsorbed on particulates, especially those particulates with size of 3.3–4.7 μm and 2.1–3.3 μm. [39].

Aldehydes are volatile organic compounds typically characterized by their irritating properties, especially the low molecular weight, the halogenated aliphatic, and the unsaturated aldehydes. In addition to irritating skin, eyes and the upper respiratory tract, aldehydes also affect nasal mucous membranes and oral passages, producing a burning sensation, bronchial constriction, choking, and coughing [41].

Exposures to formaldehyde are of concern because formaldehyde is a potent sensory irritant and is classified as a probable human carcinogen [42]. Black et al. reported that both wood dust and formaldehyde can impair mucociliary clearance [43]. Epidemiological studies have correlated wood dust and formaldehyde with nasal cancer [44,45]. Wood dust that carries formaldehyde enhances the toxicity of formaldehyde when the wood dust is intercepted and dissolved in water in the nasal cavity [46].

#### 2.5. Polycyclic aromatic hydrocarbons

The smoke emitted by incense burning has been found to contain polycyclic aromatic hydrocarbons (PAHs) [7,8,14,47-52]. In Taiwan, temples are typically heavily polluted by incense smoke, especially during special festivals, such as the Chinese New Year or the birthdays of worshiped gods. A temple was reported to have mean total-PAH concentrations of 6,258 ng/m3 and 231 ng/m^3 ^in its indoor and outdoor air, respectively; indicating that PAH concentrations of the temple's inside air were 27 times higher than that of its outside air. The top five individual PAHs having the highest concentrations (particle-bound + gas phase) were identified as acenaphthylene (3,583 ng/m^3^), naphthalene (1,264 ng/m^3^), acenaphthene (349 ng/m^3^), fluoranthene (243 ng/m^3^) and phenanthrene (181 ng/m^3^) [7]. In a study of one Swiss church, in which incense was burned, PAHs were found in sedimented dusts, indicating that incense was possibly the most significant source [53]. It also has been shown that burning incense is associated with increased levels of PAHs in homes [47,54]. In a comparison study of incense burning, Lung and Hu reported that two kinds of incense sticks generated, 17.1 ug and 25.2 ug of particle-bound PAHs, and 19.8 mg and 43.6 mg of particles per gram of incense burned, respectively [55]. It appears that different types of incense produce various amounts of PAHs.

#### 2.6. Diethylphthalate (DEP)

In India, diethylphthalate is used extensively in the incense stick industry as a binder of perfumes. It can be emitted into the air during incense burning. Eggert and Hansen reported that DEP emission from various incense could be as high as 16,365 μg/m^3 ^in concentration and 13,582 μg/unit of incense [56].

Diethylphthalate (DEP), used as a plasticizer and a detergent base, is a suspect carcinogen. Sonde et al. studied the interactive toxicity of DEP with ethyl alcohol (EtOH) in young male Sprague-Dawley rats. The rats were given 50 ppm DEP (w/v), 5% EtOH (v/v), or a combined dose of 50 ppm DEP (w/v) + EtOH (5% v/v) in water ad libitum for a period of 120 days and were maintained on normal diet. The controlled rats received normal diet and plain water. No interaction of DEP with EtOH was found. However, significantly altered lipid and enzyme levels in the liver and serum were found in the DEP-fed group. It was concluded that DEP alone leads to severe impairment of lipid metabolism coupled with toxic injury to the liver [57].

## Effects of incense smoke on airway disease and health

Like second hand smoke, pollutants emitted from incense burning in a close environment are harmful to human health. As mentioned above, particulate matters, and some of volatile organic compounds, musk ketones, musk xylenes, and musk ambrette, aldehydes, polycyclic aromatic hydrocarbons, diethylphthalate (DEP) are toxic to the lung and allergenic to the skin and eyes. While it is relatively difficult to directly study the effect of incense smoke pollutants on health, several epidemiological studies have suggested that they do cause health problems.

### 1. Airway dysfunction

Most obviously, when incense smoke pollutants are inhaled, they will cause respiratory dysfunction. In 1966, Sturton et al reported a high incidence of nasopharyngeal carcinoma in Hong Kong in male patients who burn incense as compared with the other malignant cases that were used as controls. They found that 74.5% of the studied nasopharyngeal cancer cases and 52% of all other malignant cases were exposed to incense smoke and suggested the possibility that incense smoke may be a factor in the etiology of this malignant disease [58].

In order to determine whether indoor environmental factors affected respiratory dysfunction, Yang et al. have surveyed 4,164 elementary school children in several rural areas in Kaohsiung, Taiwan. They found that, among the other chemical factors, incense burning and mosquito repellant burning were significantly associated with cough symptoms [59]. Since people working in temples may be exposed to high levels of air pollutants from incense burning, Ho et al. have investigated the prevalence of chronic respiratory symptoms and acute irritative symptoms among 109 temple workers in Kaohsiung, Taiwan. They concluded that working in a temple increases the risk for the development of acute irritative respiratory symptoms, including nose and throat irritation [60]. The adjusted odds ratios calculated for acute irritative symptoms in temple workers relative to the controls are 4.5 for throat irritation and 4.14 for nose irritation. Furthermore, chronic cough symptoms were significantly more common among the temple workers than those from the non-incense burning church, the control group.

Alarifi et al. have used rats to study the effect of incense smoke on the lung. Rats were exposed to Arabian mix incense, Ma'amoul, for 14-weeks at a rate of 4 grams/day in the exposure chamber. At the end of the exposure period, lung tissues were removed and processed for electron microscopy. It was noticed that alveolar pneumocytes of the exposed animals had significant ultrastructural changes which involved the cell organelles and surfactant material of type II cells. Neutrophil infiltration into the alveolar lumena was found to accompany degenerative and necrotic changes of the alveolar lining cells. Alveolar walls also revealed deposition of collagen fibrils which contributed in its thickening. They concluded that exposure to Ma'amoul incense could induce ultrastructural pulmonary changes which may imply compromised respiratory efficiency [61]. Similar ultrastructural pulmonary changes have also been reported in rats exposed to Bakhour, an Arabian incense [62].

It is interesting to note that in several epidemiological studies, incense burning had shown no harmful effect. In their study of the association of indoor and outdoor environmental exposures and physician-diagnosed asthma, Lee et al. surveyed 35,036 6- to 15-year-old school children in Taiwan. They reported that daily cigarette consumption in families and incense burning at home showed negative effects to the occurrence of childhood asthma. They proposed a possible explanation for their finding; cigarette smoking and incense use might have been decreased in families with children with atopic disease and thus had less atopic asthma [63]. In another study, Koo et al., analyzed data from an air pollution cross-sectional study of 346 primary school children and their 293 non-smoking mothers, and a lung cancer case-control study of 189 female patients and 197 district matched controls. They found that there was no association between exposure to incense burning and respiratory symptoms like chronic cough, chronic sputum, chronic bronchitis, runny nose, wheezing, asthma, allergic rhinitis, or pneumonia among the primary school children, their non-smoking mothers, or district matched controls. Incense burning also did not affect lung cancer risk among non-smokers, but it significantly reduced risk among smokers, even after adjusting for lifetime smoking amount. They suggested a likely explanation for this unexpected finding: incense burning was associated with certain dietary habits, i.e. more fresh fish, more retinol, and less alcohol, which have been associated with lower lung cancer risk in this population. Thus, their results indicate that diet can be a significant confounder of epidemiological studies on air pollution and respiratory health [64].

### 2. Allergy and Dermatological Effects

Lin et al. studied umbilical cord blood IgE (cIgE) in 334 mother and neonate pairs. They found that incense burning was a risk factor for elevated cIgE [65]. Lead exposure could stimulate the IgE production [66]. The concentrations of lead have been detected at 0.14 and 0.21 mg/g in PM_2.5 _and PM_2.5–10 _in the sample collected at one temple in Taiwan, respectively. It is speculated that lead emitted from incense burning could be absorbed on PM_2.5 _and PM_2.5–10 _and subsequently transferred to fetal blood and modulated the fetal immune system with IgE production. However, the authors have not yet proved the relationships between incense burning, cord blood lead, and cord blood IgE levels [65].

As indicated in the previous section, incense smoke cause morphological changes of alveolar pneumocytes and infiltration of neutrophils into alveolar lumena in experimental rats [61,62]. Activation of resident and recruited inflammatory cells can lead to elaboration of a plethora of mediators, culminating in airway inflammation and remodeling. Recent studies suggest that a dominance of the Th2 type cytokines (IL-4, IL-5, IL-10 and IL-13) may be pivotal to asthma pathogenesis [67-71]. Th2 cytokines by regulating IgE class switching as well as inducing humoral immunity, would aggravate allergic respiratory disease. While cytokines such as IL-4 and IL-13 are crucial to production of IgE by B lymphocytes, others such as IL-5 are essential to eosinophil hematopoiesis, activation and survival in tissue. Numerous factors, including incense smoke, may contribute to the development of the Th1-Th2 imbalance [72-75], and the interaction between the innate and adaptive immune systems may lead to inflammatory changes and airway remodeling [76].

Incense burning smoke has also been associated with dermatological problems. Hayakawa et al. reported a 63-year-old patient, who had practiced incense ceremony for about 15 years, and was found to have itchy depigmented macules on his dorsum manus, left shoulder and abdomen. A 48 h closed path testing revealed perfume in the incense was the cause. It was suggested that the perfume and airborne particles from the burning incense contacted the skin and caused the allergic contact dermatitis accompanied by depigmentation [77]. In addition, the same group also reported cases of contact dermatitis due to long-term exposure to musk ambrette vaporized from incense burning [78].

### 3. Neoplasm

Extracts of particulate matter from incense smoke are found to be mutagenic in the Ames Salmonella test with TA98 and activation. This suggests that incense burning can cause indoor air pollution and thus cancer akin to that from cigarette smoking [28]. To study the causes of leukemia, Lowengart et al. investigated a group of children of ages 10 years and under in Los Angeles County. The mothers and fathers of acute leukemia cases and their individually matched controls were interviewed regarding specific occupational and home exposures as well as other potential risk factors associated with leukemia. Analysis of the data from the 123 matched pairs showed an increased risk of leukemia for children whose parents burned incense at home. Furthermore, the risk was greater for more frequent users [79].

Incense smoke contains various N-nitroso compounds, which have been shown to be potent nervous system carcinogens, particularly when animals are exposed transplacentally [80]. Preston-Martin et al. studied mothers of 209 young brain tumor patients and 209 control subjects. They found that increased brain tumor risk was associated with maternal contact with nitrosamine-containing substances such as burning incense, side-stream cigarette smoke, and face makeup [81]. However, conflicting data on the effect of incense burning smoke on neoplasm have also been reported.

Several studies have shown there is no association between incense smoke and cancer. In studying risk factors associated with lung cancer in Hong Kong, Chan-Yeung et al. found that smoking was the most important risk factor associated with lung cancer, while exposure to incense smoke and frying pan fumes were not significant risk factors [82]. Similarly, McCredie et al. carried out a population-based case-control study of perinatal and early postnatal risk factors for malignant brain tumors in New South Wales children, and reported that no association was found between childhood brain tumors and incense burning [83]. A similar conclusion was reported by Koo et al. when they conducted four epidemiological studies in Hong Kong over 15 years. They found that, although incense was identified as a major source of exposure to nitrogen dioxide and airborne carcinogens, it had no effect on lung cancer risk among nonsmokers and, more intriguingly, it significantly reduced risk among the smokers [84]. They attributed the findings to the relatively healthy diets among smoking women who burned incense versus those who did not. Bunin et al. investigated risk factors for the two most common types of brain tumors in children, astrocytic glioma and primitive neuroectodermal tumor (PNET) and found that among the products (including incense) studied that contain N-nitroso compounds, only beer was associated with a significantly increased risk of either tumor type [85]. Similarly, Ger et al. investigated the relationship between various risk factors and lung cancer by histological types. They reported that, while occupational exposures to asbestos and working as a cook were significant risk factors associated with adenocarcinoma of the lung, an inverse association between incense burning and the adenocarcinoma was noted [85].

## Conclusion

Incense burning emits smoke containing particulate matter, gas products and other organic compounds and causes air pollution, airway disease and health problems. When incense smoke pollutants are inhaled, they cause airway dysfunction. Incense smoke is a risk factor for elevated cord blood IgE levels and has been indicated to cause allergic contact dermatitis. Incense smoke also has been associated with neoplasm. However, several conflicting reports have also been documented. The effect of incense smoke on health and the mechanism behind it needs to be further studied in an animal model. To obtain further conclusive results, more epidemiological studies with better controls and a longer time period are needed. Meanwhile, it is a good practice to keep the room well ventilated when burning incense. It will effectively dilute the indoor air pollutants and hence reduce the risk of exposure.

## List of abbreviations used

DEP: diethylphthalate; PAH: polycyclic aromatic hydrocarbon; PM: particulate matter; PM_10_: particulate matter less than 10 μm in diameter; VOC: volatile organic compound.

## Competing interests

The authors declare that they have no competing interests.

## Authors' contributions

T–CL, GK and DSC have all been involved in drafting the article or revising it critically for important intellectual content and have given final approval of the version to be published.
